# Identification and Expression Analysis of Putative Sugar Transporter Gene Family during Bulb Formation in Lilies

**DOI:** 10.3390/ijms25063483

**Published:** 2024-03-20

**Authors:** Ziyang Huang, Cong Gao, Yunchen Xu, Jie Liu, Jie Kang, Ziming Ren, Qi Cui, Dongze Li, Si Ma, Yiping Xia, Yun Wu

**Affiliations:** 1 Laboratory of Flower Bulbs, Department of Landscape Architecture, Zhejiang Sci-Tech University, Hangzhou 310018, China; huangziyang94@163.com (Z.H.); liujie07270907@163.com (J.L.); kangjie2570@163.com (J.K.); zimingren@zju.edu.cn (Z.R.); cuiqivivi@163.com (Q.C.); dongzeli06@163.com (D.L.); 2 Genomics and Genetic Engineering Laboratory of Ornamental Plants, College of Agriculture and Biotechnology, Zhejiang University, Hangzhou 310058, China; conggao@zju.edu.cn (C.G.); xuyunchen1998@icloud.com (Y.X.); 3 College of Horticulture, China Agricultural University, Beijing 100193, China; masi@cau.edu.cn

**Keywords:** sugar transporter, expression analysis, *Lilium*, bulblet initiation, bulblet development

## Abstract

Sugar transporters play important roles in plant growth and development, flowering and fruiting, as well as responses to adverse abiotic and biotic environmental conditions. Lilies (*Lilium* spp.) are some of the most representative ornamental bulbous flowers. Sugar metabolism is critical for bulb formation in lilies; therefore, clarifying the amount and expression pattern of sugar transporters is essential for further analyzing their roles in bulb formation. In this study, based on the transcriptome data of the *Lilium* Oriental hybrid ‘Sorbonne’ and *Lilium* × *formolongi*, a total of 69 and 41 sugar transporters were identified in ‘Sorbonne’ and *Lilium* × *formolongi*, respectively, by performing bioinformatics analysis. Through phylogenetic analysis, monosaccharide transporters (MSTs) can be divided into seven subfamilies, sucrose transporters (SUTs) can be divided into three subgroups, and sugars will eventually be exported transporters (SWEETs) can be divided into four clades. According to an analysis of conserved motifs, 20, 14, and 12 conserved motifs were predicted in MSTs, SUTs, and SWEETs, respectively. A conserved domain analysis showed that MSTs and SUTs contained a single domain, whereas most of the SWEETs harbored two MtN3/saliva domains, also known as a PQ-loop repeat. The *LohINT1*, which was predicted to have a smaller number of transmembrane structural domains, was cloned and analyzed for subcellular localization. It was found that the LohINT1 protein is mainly localized in the cell membrane. In addition, the expression analysis indicated that 22 *LohMSTs*, 1 *LohSUTs*, and 5 *LohSWEETs* were upregulated in ‘Sorbonne’ 1 day after scale detachment treatment, suggesting that they may regulate the initiation of the bulblet. A total of 10 *LflMSTs*, 1 *LflSUTs,* and 6 *LflSWEETs* were upregulated 4~6 months after sowing, which corresponds to the juvenile-to-adult transition phase of *Lilium* × *formolongi*, suggesting that they may also play a role in the accompanying bulb swelling process. Combined with quantitative real-time PCR (qRT-PCR) analysis, *LohSTP8* and *LohSTP12* were significantly overexpressed during the extremely early stage of bulblet initiation, and *LflERD6.3* was significantly overexpressed during the growth of the underground bulblet, suggesting that they may be key sugar transporters in the formation of lily bulbs, which needs further functional verification.

## 1. Introduction

Sugars are the main product of plant photosynthesis, which not only provide energy and carbon skeletons for processes such as plant growth and development and various stress responses but also play a signaling role [1,2,3]. In higher plants, sugars are mainly synthesized in source organs (such as mature leaves) and transported over long distances via the phloem to sink organs (such as plant roots, reproductive structures, storage, and development organs) that depend on nutrient supply. It is thus clear that the transportation and distribution of sugars are important for maintaining the metabolic balance between source and sink [4]. As the main form of long-distance transport of assimilates from source to sink, the loading, unloading, and distribution of sucrose are mainly dependent on the involvement of sugar transporters on the cell membrane [5,6].

The sugar transporters that have been identified in plants so far include three main classes: the monosaccharide transporters (MSTs), sucrose transporters (SUTs), and sugars will eventually be exported transporters (SWEETs) [7,8]. MSTs are Sugar_tr domain-containing members of the major facilitator superfamily (MFS) class of transporters. Structurally, MFS transporters usually contain 12 transmembrane domains (TMDs) [8,9,10]. A total of 53 MSTs were identified in the model plant *Arabidopsis* (*Arabidopsis thaliana*) and were further separated into seven subfamilies: sugar transport protein/hexose transporter (STP/HT); polyol/monosaccharide transporter (PLT/PMT); vacuolar glucose transporter (VGT); plastidic glucose transporter/suppressor of g protein beta1 (PGlcT/SGB1); tonoplastic monosaccharide transporter/tonoplast sugar transporter (TMT/TST); inositol transporter (INT); and early responsive to dehydration six-like (ERD6L). In addition, the ERD6L family includes two closely related homologous MST genes: *SFP1* (sugar-porter family protein) and *SFP2* [11,12]. These members play a variety of roles, including participating in the transport, uptake, utilization, and accumulation of monosaccharides as well as affecting sugar accumulation in plants via means such as pollen tube growth and fruiting [8,13,14]. MSTs have been identified in many species; for instance, 46 MSTs were identified in ‘Furongli’ (*Prunus salicina*) [15], 35 MSTs were identified in lotuses (*Nelumbo nucifera*) [16], 69 MSTs were identified in pears (*Pyrus bretschneideri*) [17], and 52 MSTs were identified in longans (*Dimocarpus longan*) [18]. SUTs, like MSTs, are Sugar_tr domain members of the MFS superfamily [10]. SUTs and MSTs share little homology at the amino acid level, although there are structural similarities. The structural difference between the two is usually the length of the central loop between transmembrane domains 6 and 7 [19]. According to their amino acid sequence homology and sucrose affinity, SUTs can be divided into five subgroups, among which SUT2 and SUT4 are common to monocotyledons and dicotyledons, SUT3 and SUT5 are unique to monocotyledons, and SUT1 is specific to dicotyledons [20]. SUTs play important roles in the plastid transport of sucrose and the unloading of sink organs and they have certain effects on the growth and development of plants such as flowers and fruits [21]. SUTs from a large number of plants have been identified since the discovery of SUTs in the first species; for instance, there are 9 in *Arabidopsis* [22], 5 in rice (*Oryza sativa*) [23], 5 in pears [17], 5 in petunias (*Petunia hybrida*) [24], 10 in pomegranates (*Punica granatum*) [25], and 22 in orchids (*Orchidaceae*) [26]. SWEETs, unlike MSTs and SUTs, are a newly discovered class of sugar transporters that can be bidirectional and do not rely on proton dynamic potential, characterized by containing two MtN3/saliva domains with seven transmembrane regions [7]. There are four clades of SWEETs exclusive to plants, numbered I through IV. In *Arabidopsis*, members of clade I (AtSWEET1~3) and clade II (AtSWEET4~8) transport hexose, members of clade III (AtSWEET9~15) mainly transport sucrose, whereas members of clade IV (AtSWEET16~17) are located in vesicle membranes and are mainly responsible for the transport of fructose. AtSWEET16 also transports glucose and sucrose [27,28,29]. SWEETs are involved in various physiological processes during plant growth and development, such as phloem unloading, hormone transport, pollen development, fruit development, and resistance to different stresses. Currently, SWEETs have been found in a variety of plants, including grapes (*Vitis vinifera*) [30], tomatoes (*Solanum lycopersicum*) [10], pears [31], and lychees (*Litchi chinensis*) [32].

Lilies (*Lilium* spp.) are a bulbous perennial plant belonging to Liliaceae, with high ornamental and economic value [33]. However, the low reproduction coefficient of bulbs and the long production cycle have become the bottleneck of bulb propagation, which seriously constrains the renewal, popularization, and application of new varieties of lilies [34]. Therefore, unraveling the mechanism of lily bulb formation and improving the coefficient and rate of bulb formation are crucial for improving the yield and quality of bulbous flowers. The *Lilium* Oriental hybrid ‘Sorbonne’ (abbreviated Loh) is one of the most important cut flower varieties in China, which has great market potential, but still faces the problem of bulb localization [35]. *Lilium* × *formolongi* (abbreviated Lfl), an interspecies hybrid of *Lilium longiflorum* and *Lilium formosanum*, completes the transition from its juvenile stage to adult stage in 4~6 months and is a typical short juvenile stage germplasm [36]. Juvenile-to-adult phase transition is related to bulb growth [37], which might be further utilized to improve bulb production. Our previous studies have shown that sucrose unloading mediated by cell wall invertases (CWINs) is crucial in the early stage of lily bulb formation [38]. Since the lily’s genome is large (~36 Gb) [39], no genome-wide information has been published, and the gene families of MSTs, SUTs, and SWEETs are not yet known. Therefore, in this study, based on the transcriptome data of lilies previously determined, we used bioinformatics to characterize the sugar transporter gene family of lilies; comprehensively analyzed their physicochemical properties, conserved motifs, and expression patterns; and screened out the potential key sugar transporter genes during bulb formation using qRT-PCR. These results will provide new information for us to verify the gene functions and the role of sugar transporter genes in lily bulb formation.

## 2. Results

### 2.1. Identification and Phylogenetic Analysis of the Sugar Transporters in Lilies

**Figure 1:**
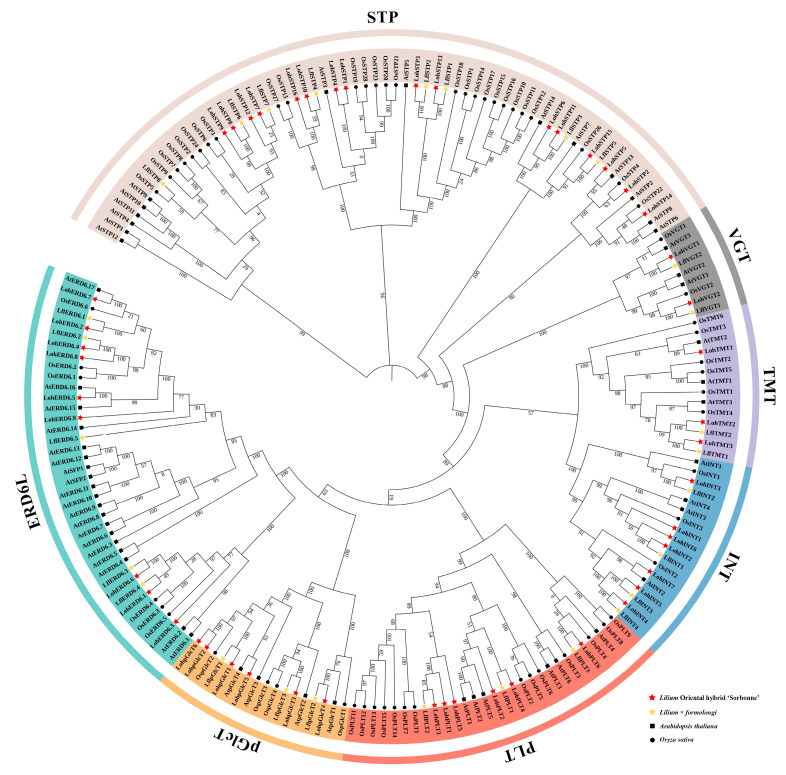
Phylogenetic analysis of MSTs in lilies, *Arabidopsis*, and rice. The sequences of 191 MSTs of ‘Sorbonne’, *Lilium* × *formolongi*, *Arabidopsis thaliana*, and *Oryza sativa* were aligned using the MUSCLE Wrapper tool, and a phylogenetic tree was constructed using the FastTree maximum likelihood (MJ) method. STP, sugar transport protein; VGT, vacuolar glucose transporter; TMT, tonoplastic monosaccharide transporter; INT, inositol transporter; PLT, polyol transporter; pGlcT, plastidic glucose transporter; ERD6L, plastidic glucose transporter.

Based on the preliminary HMM search and Blastp comparison as well as the validation and de-redundancy of the sequences, a total of 49 LohMSTs, 5 LohSUTs, and 15 LohSWEETs were finally identified in the ‘Sorbonne’ transcriptome, and 27 LflMSTs, 2 LflSUTs, and 12 LflSWEETs in the *Lilium* × *formolongi* transcriptome. The identified lily sugar transporters were renamed according to the previous studies on *Arabidopsis*, and a phylogenetic tree was constructed together with the homologous sequences of sugar transporters from *Arabidopsis* and rice. The MST family can be divided into seven independent subfamilies. Among the 49 MSTs identified in ‘Sorbonne’, STP contains 16 members, ERD6L contains 9 members, and INT, pGlcT, PLT, TMT, and VGT contain 7, 6, 6, 3, and 2 members, respectively. Among the 27 MSTs identified in *Lilium* × *formolongi*, STP remained the subfamily with the most MSTs, which contains eight members, and the subsequent subfamilies in descending order of the number of members were ERD6L, INT, pGlcT, PLT, TMT, and VGT, with five, four, three, three, two, and two members, respectively. It is evident that STP and ERD6L constitute the two largest branches of MSTs of the lilies used (Figure 1). The amino acid lengths of a total of 76 MSTs in ‘Sorbonne’ and *Lilium* × *formolongi* ranged from 203 aa (LohERD6.3) to 753 aa (LflTMT1), the number of transmembrane regions ranged from 1 (LohTMT1) to 13 (LohSTP7), and the isoelectric points ranged from 4.80 (LohTMT1) to 10.19 (LflSTP2); the cell membrane is the preferred subcellular localization for all MSTs, in addition to which LohTMT1 may also localize to the nucleus (Table 1).

**Figure 2:**
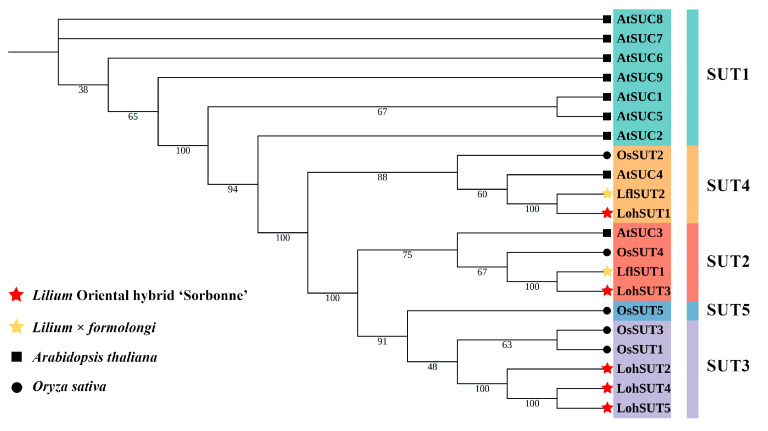
Phylogenetic analysis of SUTs in lilies, *Arabidopsis*, and rice. The sequences of 21 SUTs of ‘Sorbonne’, *Lilium* × *formolongi*, *Arabidopsis thaliana,* and *Oryza sativa* were aligned using the MUSCLE Wrapper tool, and a phylogenetic tree was constructed using the FastTree maximum likelihood (MJ) method.

In agreement with previous reports, the SUTs of lilies, as monocotyledons, were distributed in the SUT2 and SUT4 subgroups common to monocotyledons and dicotyledons and the SUT3 subgroup specific to monocotyledons, whereas the SUT5 subgroup was not found to be distributed in our transcriptome, which may be related to the fact that they are not expressed during the biological process tested. Among the five SUTs identified in ‘Sorbonne’, LohSUT3 was located in the SUT2 subgroup, LohSUT1 in the SUT4 subgroup, and LohSUT2, LohSUT4, and LohSUT5 in the SUT3 subgroup. The two SUTs, LflSUT1 and LflSUT2, identified in *Lilium* × *formolongi*, on the other hand, were located in the SUT2 and SUT4 subgroups, respectively (Figure 2). The amino acid lengths of a total of seven SUTs from ‘Sorbonne’ and *Lilium* × *formolongi* ranged from 213 aa (LohSUT2) to 590 aa (LohSUT3), the number of transmembrane regions ranged from 5 to 12, and the isoelectric points ranged from 6.74 (LflSUT1) to 9.40 (LohSUT5), with subcellular localization all located at the cell membrane (Table 2).

**Figure 3:**
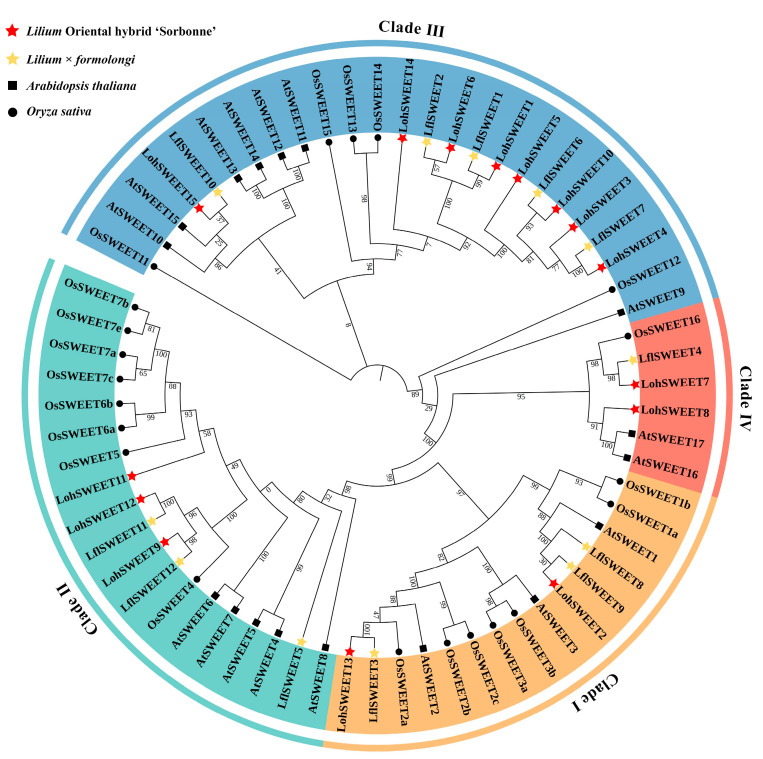
Phylogenetic analysis of SWEETs in lilies, *Arabidopsis*, and rice. The sequences of 65 SWEETs of ‘Sorbonne’, *Lilium* × *formolongi*, *Arabidopsis thaliana,* and *Oryza sativa* were aligned using the MUSCLE Wrapper tool, and a phylogenetic tree was constructed using the FastTree maximum likelihood (MJ) method.

Based on the phylogenetic analysis, SWEETs of ‘Sorbonne’ and *Lilium* × *formolongi* could be divided into four different clades. Clade I contained two LohSWEETs and three LflSWEETs. Clade II contained three LohSWEETs and three LflSWEETs each. Clade III clustered the highest number of SWEETs, containing eight LohSWEETs and five LflSWEETs, while clade IV contained only two LohSWEETs and one LflSWEET (Figure 3). Of the 27 SWEETs predicted for ‘Sorbonne’ and *Lilium* × *formolongi*, the amino acid lengths ranged from 77 aa (LflSWEET10) to 331 aa (LohSWEET6), the number of transmembrane regions ranged from one to seven, and the isoelectric points ranged from 5.29 (LohSWEET6) to 10.01 (LflSWEET8), with most of the subcellular localizations at the cell membrane and a few at the chloroplasts (LohSWEET11, LohSWEET15, LflSWEET4, LflSWEET5, LflSWEET10) and the peroxisomes (LflSWEET4) (Table 3).

### 2.2. Analysis of Conserved Motifs and Domains of Lily Sugar Transporters

**Figure 4:**
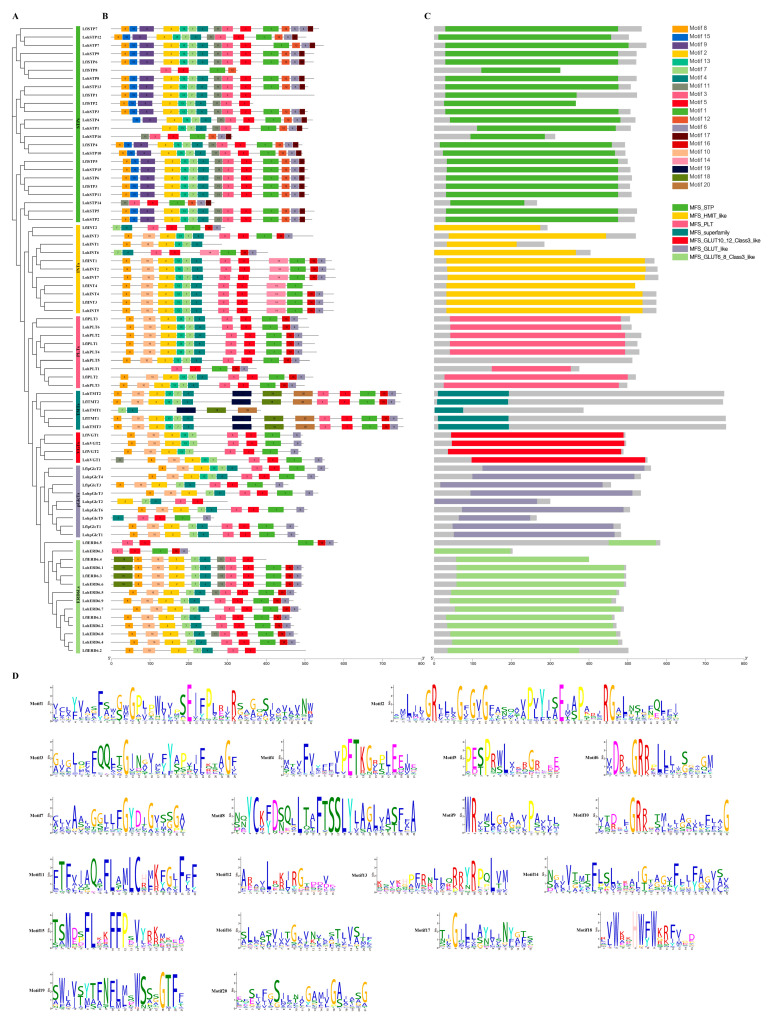
Phylogenetic relationships, conserved motifs, and conserved domain analysis of ‘Sorbonne’ and *Lilium* × *formolongi* MSTs. (**A**) Phylogenetic trees of LohMSTs and LflMSTs were constructed using the maximum likelihood method. Seven subfamilies were labeled. (**B**) Motif compositions of LohMSTs and LflMSTs. A total of 20 motifs are shown as rectangles with different colors. (**C**) Domain compositions of LohMSTs and LflMSTs. (**D**) Amino acid sequences of the 20 conserved motifs of LohMSTs and LflMSTs are shown.

**Figure 5:**
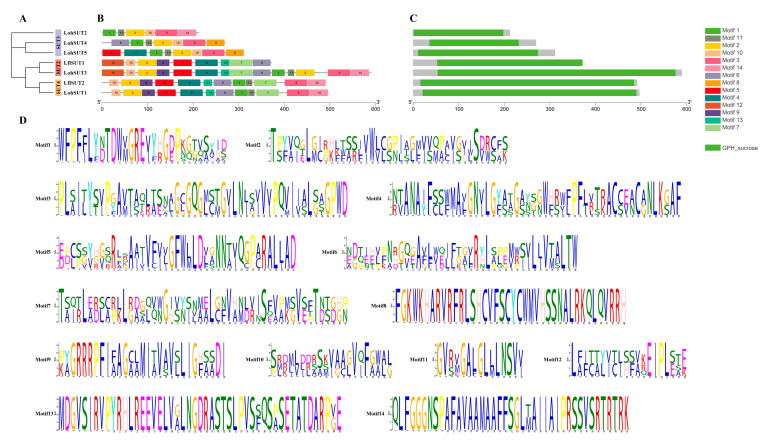
Phylogenetic relationships, conserved motifs, and conserved domain analysis of ‘Sorbonne’ and *Lilium* × *formolongi* SUTs. (**A**) Phylogenetic trees of LohSUTs and LflSUTs were constructed using the maximum likelihood method. Three subgroups were labeled. (**B**) Motif compositions of LohSUTs and LflSUTs. A total of 14 motifs are shown as rectangles with different colors. (**C**) Domain compositions of LohSUTs and LflSUTs. (**D**) Amino acid sequences of the 14 conserved motifs of LohSUTs and LflSUTs are shown.

**Figure 6:**
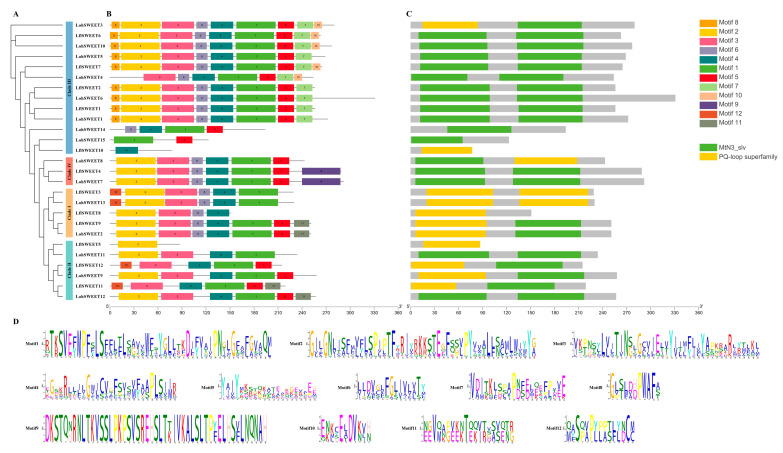
Phylogenetic relationships, conserved motifs, and conserved domain analysis of ‘Sorbonne’ and *Lilium* × *formolongi* SWEETs. (**A**) Phylogenetic trees of LohSWEETs and LflSWEETs were constructed using the maximum likelihood method. Four clades were labeled. (**B**) Motif compositions of LohSWEETs and LflSWEETs. A total of 12 motifs are shown as rectangles with different colors. (**C**) Domain compositions of LohSWEETs and LflSWEETs. (**D**) Amino acid sequences of the 12 conserved motifs of LohSWEETs and LflSWEETs are shown.

After that, we analyzed the conserved motifs of lily sugar transporters through the MEME server. A total of 20 conserved motifs were predicted in the MSTs. Motif3 was present in almost all MSTs, indicating that it is important in lily MSTs. Motif9, motif15, and motif17 were only present in the STP subfamily. Motif14 was only present in the INT subfamily, suggesting that they may be necessary for STP and INT subfamilies, respectively. Conserved domain analysis showed that all structurally similar members clustered in the same subfamily (Figure 4). A total of 14 conserved motifs were identified for SUTs. Motif2 and motif10 were present in all SUTs, indicating that they are conserved domains of SUTs. Although MSTs and SUTs have the same transmembrane domains according to previous reports, the conserved motifs between them are quite different, suggesting that MSTs and SUTs are functionally distinct from each other (Figure 5). A total of 12 conserved motifs were identified in SWEETs. Motif1 was present in almost all SWEETs. Motif7, motif8, and motif10 were present only in clade III, and motif9 was unique to clade IV. In addition, the protein sequences of members of the lily SWEETs family are relatively conserved, with most SWEETs containing two MtN3/slv domains (CDD accession No. pfam03083) or the PQ-loop superfamily (CDD accession No. pfam03083) in similar positions, whereas the smaller portion of SWEETs (LohSWEET14, LohSWEET15, LflSWEET10, LflSWEET8, and LflSWEET5) had only one MtN3/slv domain or PQ-loop superfamily, which is possibly due to the fact that all of these sequences were derived from lily unigenes rather than full-length genes (Figure 6)

### 2.3. Cloning and Subcellular Localization Analysis of LohINT1 Gene

**Figure 7:**
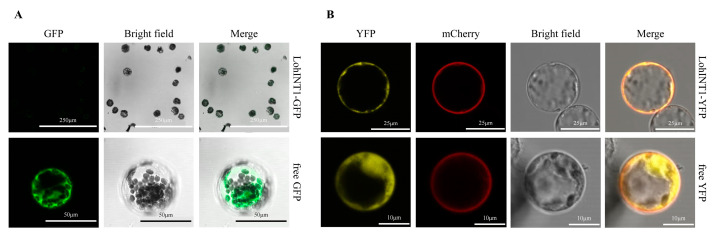
Subcellular localization analysis of LohINT1. (**A**) Subcellular localization of LohINT1-GFP and free GFP. (**B**) Subcellular localization of LohINT1-YFP and free YFP.

To verify the robustness of the transcriptome data, we selected the *LohINT1* gene (6TMDs) with a small number of TMDs as an example of MSTs to carry out cloning and subcellular localization analysis. Firstly, a *LohINT1*-specific band was obtained under 1% agarose gel electrophoresis analysis (Figure S1A), and the sequencing result was highly consistent with the original sequence from the transcriptome. To examine the subcellular localization of the LohINT1 protein, the open reading frame (ORF) of the *LohINT1* gene was fused to the N-terminal of the GFP reporter, driven by the CaMV35S promoter. Both the recombined (LohINT1-GFP) and unrecombined (free GFP) vectors were transferred into maize yellowing seedling protoplasts. The results showed that LohINT1-GFP subcellularly localized without a signal (Figure 7A). The experiments were carried out several times to exclude the technical issues and still no signal could be observed. We, therefore, proposed that the ORF might be incomplete as the predicted protein is only 285 aa in length while its orthologous gene in *Arabidopsis thaliana* is 582 aa in length. We then carefully checked the original sequence in the NCBI database by the basic local alignment search tool (BLAST) and found a base deletion (T) probably resulting the early termination of the protein.

Afterwards, by designing the primer according to the new coding sequence, we amplified *LohINT1* using the previously obtained ‘Sorbonne’ complementary DNA (cDNA) as a template (Figure S1B). The sequencing results revealed that the total length of the *LohINT1* gene is 1743 bp, encoding 580 amino acids. The recombinant LohINT1-YFP was constructed with mCherry-labeled cell membranes as a marker. The results showed that LohINT1-YFP was mainly localized in the cell membrane, with a small distribution in other endomembrane systems (Figure 7B). Based on the above-mentioned observations, we speculated that the low number of TMDs contained in some sugar transporters may also be due to incomplete transcriptome data.

### 2.4. Expression Patterns of Sugar Transporter Genes at Different Stages of Lily Bulb Initiation and Development

**Figure 8:**
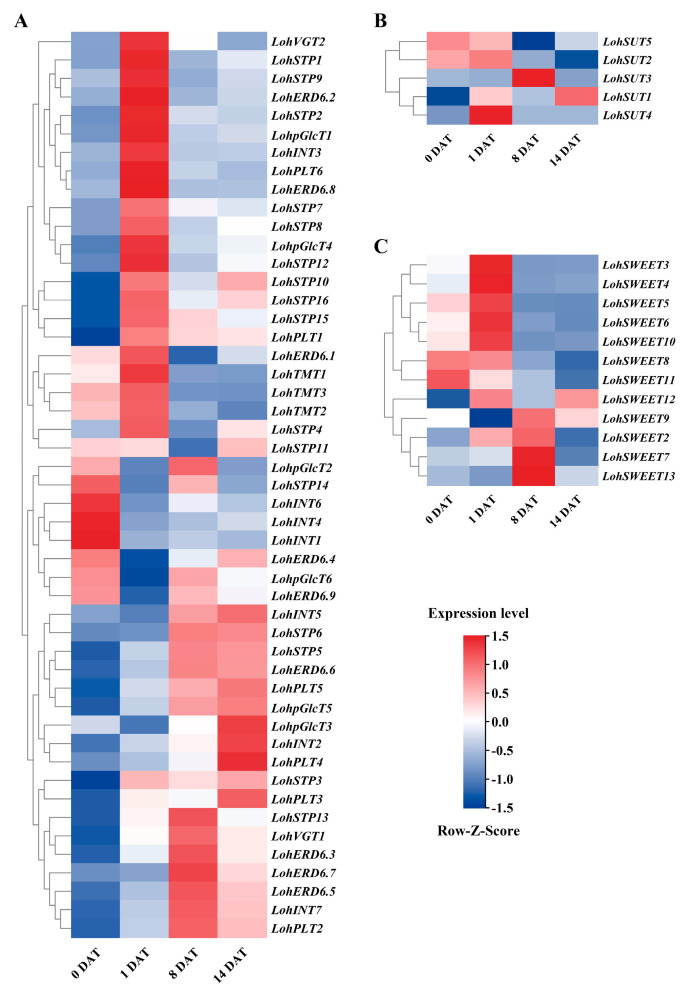
Expression patterns of ‘Sorbonne’ sugar transporter genes at four periods under aeroponic conditions. (**A**) Expression patterns of *LohMSTs* during four periods of bulblet initiation. (**B**) Expression patterns of *LohSUTs* during four periods of bulblet initiation. (**C**) Expression patterns of *LohSWEETs* during four periods of bulblet initiation. Color scale represents reads per kilobase per million normalized log^2^ transformed counts, where dark red indicates high level, dark blue indicates low level, and white indicates medium. DAT, days after treatment.

**Figure 9:**
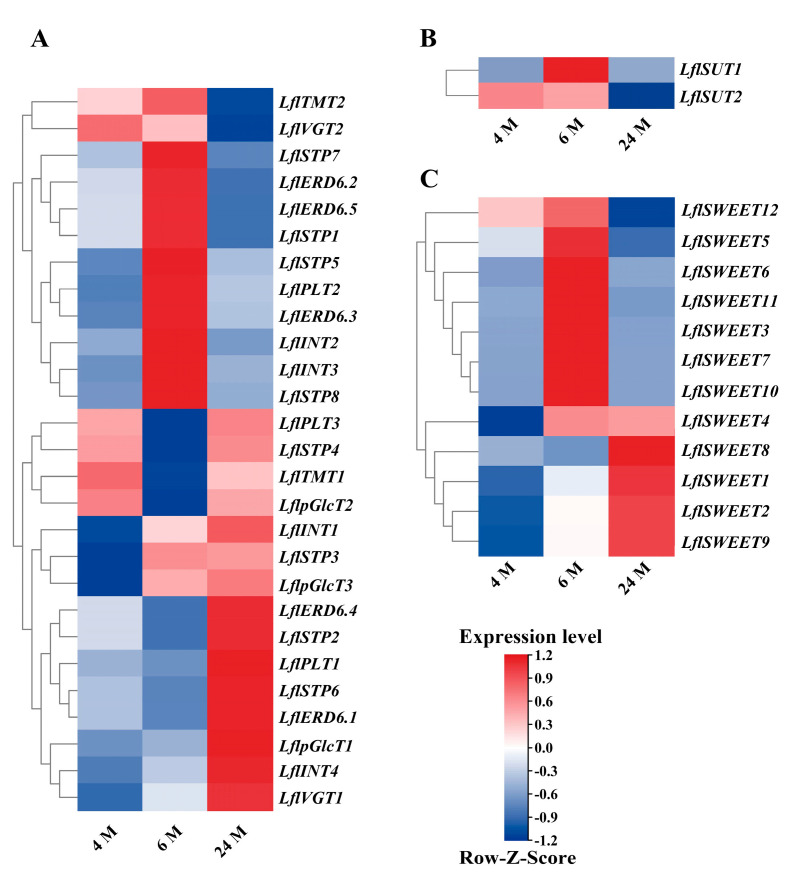
Expression patterns of *Lilium* × *formolongi* sugar transporter genes at three periods after sowing. (**A**) Expression patterns of *LflMSTs* at three periods during the growth of the underground bulblet. (**B**) Expression patterns of *LflSUTs* at three periods during the growth of the underground bulblet. (**C**) Expression patterns of *LflSWEETs* at three periods during the growth of the underground bulblet. Color scale represents reads per kilobase per million normalized log^2^ transformed counts, where dark red indicates high level, dark blue indicates low level, and white indicates medium. M, months.

Based on the expression of transcriptome data, we mapped the expression patterns of different sugar transporter genes during bulb initiation and development and clustered them logarithmically. The expression of sugar transporter genes at the stage of bulblet initiation was observed by aeroponic ‘Sorbonne’ scales. The outer scale from the mother bulblet was applied detachment treatment and the previous studies in the laboratory divided the process of lily bulb formation into four key stages: the stage of scale detachment (0 days after treatment (DAT)), the stage of wound response and early regeneration competence (1 DAT), the stage of adventitious bud initiation (8 DAT), and the stage of adventitious bud swelling and bulblet formation (14 DAT). Among them, 1 DAT is the early stage of ontogeny and is critical for bulblet initiation, so this stage was chosen to explore the situation of sugar transporter-related genes. The results showed that 22 *LohMSTs*, 1 *LohSUTs*, and 5 *LohSWEETs* were upregulated about 1.5-fold at 1 DAT (Figure 8). Similarly, based on previous studies, the bulb swelling and development process could be divided into three main stages: the juvenile stage (2~4 months (M)), the transition stage (4~6 M), and the adult stage (6~24 M). We focused on the expression of sugar transporter genes during bulb swelling accompanied by the simultaneous transition stage of lilies by sampling the shoot apical meristem (SAM) of *Lilium × formolongi* at 4 M, 6 M, and 24 M after sowing. Sugar transporter genes upregulated at 4~6 M and downregulated at 6~24 M were highlighted, which contained 10 *LflMSTs*, 1 *LflSUTs,* and 6 *LflSWEETs*; they were upregulated nearly 2-fold around 4~6 M (Figure 9).

### 2.5. Validation of Lily Sugar Transporter Genes Expression by qRT-PCR

**Figure 10:**
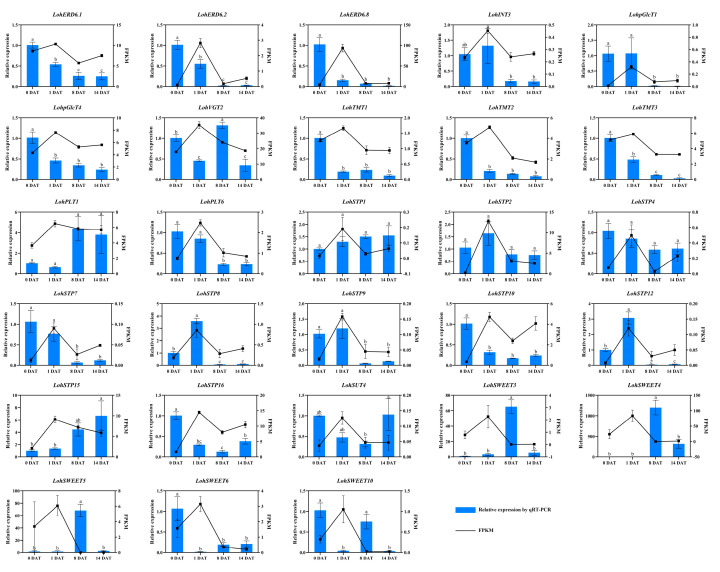
Expression profiles of 28 *LohSTs* genes during the extremely early stage of bulblet initiation. All data are presented as mean ± standard error of mean (SEM). Lowercase letters above the bars indicate significant differences between periods (*p*-value < 0.05, LSD, Duncan).

**Figure 11:**
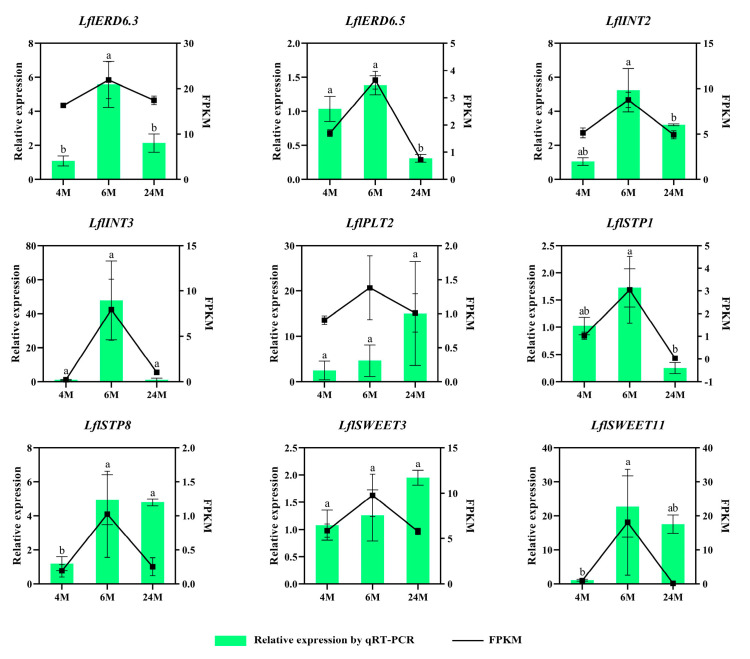
Expression profiles of 9 *LflSTs* genes during the growth of the underground bulblet. All data are presented as mean ± SEM. Lowercase letters above the bars indicate significant differences between periods (*p*-value < 0.05, LSD, Duncan).

The expression levels of some genes associated with the initial and developmental process of lily bulbs were chosen and analyzed by qRT-PCR, and the quantitative analysis results were compared with the expression trends of FPKM (fragments per kb per million) values. In ‘Sorbonne’ we verified all 28 genes (Figure 10), and the expression of two sugar transporter genes, *LohSTP8* and *LohSTP12*, were upregulated and expressed at least 3-fold at 1 DAT, which was significantly higher than at the other stages, and this was consistent with the results by the FPKM expression pattern. The qRT-PCR analysis of other genes showed that the expression levels of *LohERD6s*, *LohTMTs*, *LohpGlcT4*, *LohSTP10,* and *LohSWEET6* decreased gradually from 0 DAT. The expression levels of *LohVGT2*, *LohSTP16,* and *LohSWEET10* were significantly lower at 1 DAT. The expression levels of *LohSWEET3*, *LohSWEET4,* and *LohSWEET5* were significantly increased at 8 DAT. The expression levels of *LohSTP15* and *LohSUT4* were significantly increased at 14 DAT. There were no significant differences in the expression levels of *LohINT3*, *LohpGlcT1*, *LohPLT6*, *LohSTP7,* and *LohSTP9* between 0 DAT and 1 DAT, while *LohPLT1*, *LohSTP1*, *LohSTP2,* and *LohSTP4* were not significantly different at any of these four stages. Some genes were selected for validation in *Lilium* × *formolongi* (Figure 11). The qRT-PCR results showed that one sugar transporter gene, *LflERD6.3*, was significantly overexpressed at 4~6 M and upregulated about 5.5-fold, which was also in line with the results of the FPKM expression pattern. In addition, *LflINT2*, *LflERD6.5,* and *LflSTP1* showed no significant change at 4~6 M and then decreased significantly at 6~24 M. On the contrary, *LflSTP8* and *LflSWEET11* were significantly elevated at 4~6 M and had no significant change at 6~24 M. *LflINT3*, *LflSTP12*, and *LflSWEET3* had no significant differences.

## 3. Discussion

The initiation and development of bulbs are crucial to the growth cycle of the lily, which will further affect the yield and quality of the lily [34]. As an important storage organ, the bulb of a lily mainly accumulates substances through starch synthesis, by which the decomposition and transport of sucrose provide important precursor substances for the synthesis of starch [40]. Previous studies have shown that sucrose is the main component of phloem transport of lilies [41]; so, sucrose metabolism, especially sucrose unloading, plays an important role in carbon allocation during the initiation and development of lily bulbs. The main ways through which sucrose enters the sink cells are the symplastic pathway and the apoplastic pathway. Among them, the sucrose in the apoplastic pathway is transported by SWEETs located in the cell membrane, and then unloaded directly to the cytoplasm via SUTs, or hydrolyzed to glucose and fructose by CWINs and then transported to the cytoplasm via MSTs. It can be seen that sugar transporters are key substances in the apoplastic unloading pathway [40,42,43].

Previous studies of sugar transporter genes in the allocation of assimilates of *Lilium* Oriental hybrid ‘Sorbonne’ have been carried out to observe the assimilates’ allocation by determining the carbohydrate contents in different tissues of five critical stages during lily development, including the bulb setting stage, the plant height of 30 cm with leaf-spread stage, the budding stage, the flowering stage, and the final flowering stage. Finally, three sugar transporter genes that play key roles in the accumulation and transportation of assimilates in lilies were further identified among 16 sugar transporter genes related to sugar transport and metabolism [44]. Additionally, the importance of carbohydrates during flowering has also been explored through the study of ‘Sorbonne’, which mainly focused on the effect of *SUT* genes [45]. Unlike the previous biological processes from bulb sowing to flowering and vernalization to flower bud vernalization, the biological process we focused on is the initial and developmental stages of lily bulblets. Our systematic study will provide a basis for the functional validation and further investigation of the mechanism of action by mining the candidate genes related to the bulb formation process.

In this study, 69 sugar transporters were identified in the ‘Sorbonne’ transcriptome, including 49 MSTs, 5 SUTs, and 15 SWEETs. Likewise, 41 sugar transporters, including 27 MSTs, 2 SUTs, and 12 SWEETs, were identified in the *Lilium* × *formolongi* transcriptome. According to the identification of different sugar transporter families, MSTs tended to contain the largest number of gene family members, while SUTs belonged to a relatively small gene family, which may be related to the fact that MSTs contained more subfamilies and could transport more types of sugars. Similar results have been observed in other plants. For example, 69 MSTs and 6 SUTs were found in pears [17], 64 MSTs and 9 SUTs were found in apples (*Malus domestica*) [46], and 46 MSTs and 6 SUTs were found in longans [18]. The number of SWEETs was usually intermediate between MSTs and SUTs. Phylogenetic analysis showed that both LohMSTs and LflMSTs could be divided into seven independent subfamilies, and STP and ERD6L constituted the two largest branches of the MSTs (Figure 1), which is consistent with previous reports on other plants, such as strawberries (*Fragaria* × *ananassa*) [47], longans [18], lotuses [16], jujubes (*Ziziphus jujuba*) [48], etc. Most of the LohSUTs and LflSUTs are highly homologous with rice and were distributed on SUT2, SUT3, and SUT4, where LflSUTs were not found in SUT3. LohSWEETs and LflSWEETs were divided into four clades (clade I to clade IV), which is consistent with *Arabidopsis* [7], rice [49], grapes [50], lychees [32], daylilies (*Hemerocallis citrina*) [51], etc. In addition, SWEETs aggregated in clade III are the most numerous, similar to the results for lychees [32], bananas (*Musa acuminata*) [52], and alfalfa (*Medicago truncatula*) [53]. It is worth noting that a batch of sugar transporter genes (18) has already been reported in ‘Sorbonne’ during the process of vernalization and flowering [44,45], and our results would be a fine supplement to this.

Conserved motif analysis showed that different sugar transporter families all contained some essential conserved motifs, which is consistent with the results for pears [17], longans [18], and peppers (*Capsicum annuum*) [54], suggesting that they are of special significance for different sugar transporters. These results also imply that they have different functions in different clades of different sugar transporters in lilies. At the same time, some studies have found that those with similar motifs in general not only belong to a subfamily but also may be correlated in their biological functions [55]. As for the situation that some motifs were not included in some sequences despite the significant level of conservation, we speculate that there may be two reasons; the first one may be that some motifs were missing due to incomplete transcriptome data, and such sequences were retained because they still have conserved domains that can validate them as sugar transporters despite the incomplete motifs included in the sequences. We also confirmed this speculation by subcellular localization analysis. The second possibility is that many sugar transporters have undergone TMD deletion events at the N-terminal and C-terminal during evolution. In terms of conserved domains, MFS transporters in plants possess a common structure of 12 transmembrane domains (TMD1–TMD12), which are separately contained within the N-terminal (TMD1–TMD6) and the C-terminal (TMD7–TMD12), and each of these domains contains five to seven transmembrane-spanning *α* helices, with six being the most common number [56,57,58]. In the current study, 10 LohMSTs, 8 LflMSTs, and 3 LohSUT, 1 LflSUT had fewer than 10 TMDs. Consistent with this, similar protein structures are also observed in tomatoes [10] and grapes [30]. A related speculation is that the loss of the N-terminal or C-terminal regions may have occurred in some MSTs and SUTs during evolution. Taking *LohINT1*, an MST gene containing fewer TMDs, as an example for clonal sequencing and subcellular localization, its sequencing results were highly consistent with the transcripts, but there was no signal for subcellular localization, and further amplification of the full length of *LohINT1* sequence showed that its subcellular localization was mainly located in the cell membrane, which was in agreement with the previous prediction in Table 1. This verified that the transcriptome data were not completely reliable. It suggests that the low number of TMDs in some MSTs and SUTs may be due to incomplete transcriptome data (Table S4). However, a low number of TMDs still possibly exists as reported in [53,59]. It is impossible to check all the incomplete sequences experimentally in the current study. We recommend further BLAST to check the base deletion/insertion or the rapid amplification of cDNA ends method could be considered to obtain the full-length sequence when verifying the upstream biological function for those genes marked as incomplete in Table S4; the original sequence has been shown in Table S5. The same situation may also exist in SWEETs, making the number of TMDs in some SWEETs fewer than seven. Notably, 13 TMDs were predicted in LohSTP7, which may arise from the duplication of adjacent genes [9]. Comparing different sugar transporters, although MSTs and SUTs have relatively similar domains, the conserved motifs of the two are quite different, indicating that MSTs and SUTs play different functions in the process of sugar transport. Furthermore, among MSTs, different subfamilies have similar single domains, indicating that members of the same subfamily have the same function. In contrast, the domains contained in SWEETs are relatively conservative, with most of them containing two MtN3/slv domains (also known as a PQ-loop repeat), which also implies that SWEETs have functional diversity during plant growth and development.

Sugar transporter genes constitute a versatile gene family, and they play critical roles in many biological processes during plant growth and responses to environmental stimuli [7,12]. *LoSWEET14* is a sugar transporter that has been identified in recent years as potentially involved in the abscisic acid (ABA) signaling pathway to regulate sugar accumulation under abiotic stresses in lilies [60]. In our study, the counterpart to *LoSWEET14* is *LohSWEET4*, which has >90% sequence similarity and was significantly expressed during bulblet initiation 8 days after aeroponics, which suggests *LohSWEET4* might be crucial for adventitious bud initiation and needs further verification. By contrast, we are most interested in genes that play important roles in the formation of lily bulbs. In ‘Sorbonne’, 28 sugar transporter genes (including 22 *LohMSTs*, 5 *LohSUTs*, and 5 *LohSWEETs*) were upregulated at the stage of wound response and early regeneration competence, indicating that starch accumulation and sucrose metabolism are active in this bulblet initiation stage, and these genes may also be relevant to bulb formation. STPs are responsible for the transport of monosaccharides and proton cotransport into the cell and also play an important role in sugar transport in *Arabidopsis* and rice [14,61]. Apple MdSTP13a regulates apple pollen tube growth by taking up both hexose and sucrose [62]. *Lupinus polyphyllus* LpSTP1 can transport a variety of hexose substrates [63]. In this study, 10 of the 22 *LohMSTs* upregulated at the stage of wound response and early regeneration competence belonged to STPs. After a subsequent qRT-PCR verification, *LohSTP8* and *LohSTP12* were significantly overexpressed during the initiation of small bulblets. Phylogenetic relationships revealed that *LohSTP8* and *LohSTP12* are most closely related to *AtSTP1*, which is mainly expressed in germinating seeds, young seedlings, and guard cells and mainly mediates the transport of monosaccharides such as hexose and glucose in addition to fructose [64]. Therefore, it can be speculated that ‘Sorbonne’ *LohSTP8* and *LohSTP12* may be involved in the transport of monosaccharides after unloading the hydrolysis of sucrose in the apoplastic pathway. Seventeen sugar transporter genes (including *LflMSTs10*, *LflSUTs1*, and *LflSWEETs6*) were upregulated at the transition stage in *Lilium* × *formolongi*, indicating that there possibly may be a transition in this stage where sucrose was unloaded from the symplast pathway to the apoplast pathway, and these genes may also be involved in the growth of the underground bulblet. ERD6L is one of the least studied subfamilies with very few members characterized. It has been shown to be involved in keeping a balance of glucose between the inside and outside of the vacuole [65]. In this study, 3 of the 10 *LflMSTs* upregulated at the transition stage belonging to ERD6L. *LflERD6.3* was significantly overexpressed during the bulb swelling that accompanies the transition of lilies from juvenile to adult after verification by qRT-PCR, suggesting that this gene may be involved in bulb carbohydrate accumulation. As a result, *LohSTP8*, *LohSTP12,* and *LflERD6.3* were selected as key sugar transporter genes during bulb formation in lilies for subsequent functional verification.

## 4. Materials and Methods

### 4.1. Plant Material and Culture Conditions

*Lilium* Oriental hybrids of ‘Sorbonne’ and *Lilium* × *formolongi* were selected as materials to investigate the process of lily bulb initiation and development by using aeroponics and potted planting, respectively. The bulbs of ‘Sorbonne’ were cleaned and sterilized by soaking in carbendazim (1:1000) for 30 min before aerial cultivation, and the outer 1~2 layers of full scales were peeled off from the basal plate and laid on 3~4 layers of moist sterilized gauze near the axial surface, and then they were placed on a plastic tray within a plant growth chamber in Zhejiang University (118°21′–120°30″, 29°11′–30°33″) with temperatures at 24 °C, humidity at 90–95%, and light/darkness = 12/12 h. Plump and uniform seeds of *Lilium* × *formolongi* were sown aseptically and then propagated in seed media (4.43 g/L MS, 1.0 mg/L 6-BA, 0.2 mg/L NAA, 30 g/L Sucrose, 3 g/L Phytagel, PH 5.8) at (24 ± 2) °C. After 15 days of dark culture, germinated seeds with strong growth consistency were selected and transferred to 50-well cavity trays with temperatures of 24 °C. After about two months of growth, they were transferred to planting pots with temperatures of 24 °C, 16/8 h of light/darkness, and a light intensity of 80 μmoL m^−2^s^−1^ PPFD. The seedling medium of the basin plate was peat: perlite: vermiculite = 2:1:1 (*v*/*v*/*v*). The samples were selected from the critical periods of bulbs initiation and development, sampling points included 0 DAT, 1 DAT, 8 DAT, and 14 DAT under the condition of ‘Sorbonne’ aerial cultivation and 4 M, 6 M, 24 M after the sowing of *Lilium* × *formolongi*. All samples were rapidly frozen with liquid nitrogen and stored at −80 °C for subsequent analysis, and three biological replicates were carried out for each treatment to ensure that the data were accurate and reliable.

### 4.2. Identification of Sugar Transporters

The transcriptome data of ‘Sorbonne’ and *Lilium* × *formolongi* were obtained by the previous sequencing of our research group, and the library for ‘Sorbonne’ is a merged one including PacBio full-length sequencing (Table S6). To identify lily sugar transporters, the HMMER profiles of Sugar_tr domain (PF00083), MFS-1 (PF07690), MFS-2 (PF13347), and MtN3_slv (PF03083) were first downloaded from Pfam (http://pfam.xfam.org/, accessed on 9 April 2023) [66]. The hmmsearch command in TBtools version 2.0 was used to detect the ‘Sorbonne’ and *Lilium* × *formolongi* transcriptome databases [67]. The search results were manually checked to remove the redundancy initially, where E-value < 1 × 10^−3^. The *Arabidopsis* sugar transporters sequences were downloaded from the TAIR website (https://www.arabidopsis.org/, accessed on 9 April 2023) and used as query sequences to perform Blastp sequence comparison to find the best matching sequences and remove redundancy initially.

The above candidate sequences were validated by online analysis using the NCBI conserved domain database (CDD) (https://www.ncbi.nlm.nih.gov/Structure/bwrpsb/bwrpsb.cgi, accessed on 9 April 2023) [68] and selected Uniprot for reverse Blastp in the NCBI BLAST (https://blast.ncbi.nlm.nih.gov/Blast.cgi, accessed on 9 April 2023) to comprehensively identify whether they have the family’s characteristic domains. Sequences that do not contain conserved domains were removed; similar sequences were clustered to remove redundancies using the CD-HIT web server (https://www.bioinformatics.org/cd-hit/, accessed on 10 April 2023) with the parameters set as identity 0.9, threads 10, and word_length 5 [69]; and finally, the sequences of lily sugar transporters family members were obtained.

### 4.3. Phylogenetic Analysis

A multiple sequence comparison of the identified lily sugar transporters with those of *Arabidopsis* and rice was performed using the MUSCLE Wrapper tool in the TBtools version 2.0 (Table S1). Phylogenetic trees were constructed using the maximum likelihood (MJ) method in FastTree version 10.0.19045.4170 [70], and the constructed evolutionary trees were collapsed and formatted with using the online tool iTOL version 6 (https://itol.embl.de/, accessed on 12 April 2023) [71].

### 4.4. Physicochemical Property Analysis and Prediction of Subcellular Localization

Properties such as isoelectric point (pI), protein molecular weight (MW), and other attributes of the obtained protein sequences were predicted using the online analysis tool EXPASY ProtParam (https://web.expasy.org/protparam/, accessed on 15 April 2023) [72]. Signal peptide prediction was accomplished using SignalP-4.1 (https://services.healthtech.dtu.dk/services/SignalP-4.1/, accessed on 15 April 2023) [73]. The number of transmembrane regions was predicted using TMHMM2.0 (https://services.healthtech.dtu.dk/services/TMHMM-2.0/, accessed on 15 April 2023) [74]. The proteins encoded by candidate genes were analyzed for subcellular localization prediction using the online Plant mPLoc website (http://www.csbio.sjtu.edu.cn/bioinf/plant-multi/, accessed on 15 April 2023) [75]. Protein secondary structure was predicted using SOPMA (https://npsa-prabi.ibcp.fr/cgi-bin/npsa_automat.pl?page=npsa_sopma.html, accessed on 15 April 2023) [76].

### 4.5. Conserved Domain Analysis and Motif Distribution Analysis

Motif prediction of proteins was performed using MEME Suite version 5.5.3 (https://meme-suite.org/meme/, accessed on 17 April 2023) [77], and the conserved regions of the key conserved domains were preserved. All parameter settings were set as default, except the maximum number of predicted motifs which was set to 20, and the motif distribution was plotted in combination with TBtools version 2.0.

### 4.6. Cloning and Subcellular Localization Analysis of LohINT1 Gene

Taking MSTs as an example, based on the number of TMDs of the 76 MSTs identified, LohINT1 (6TMDs) was selected from the proteins with the number of TMDs fewer than 10 for clone sequencing and subcellular localization analysis. The transcript DNA (cDNA) of the LohINT1 gene was extracted from the ‘Sorbonne’ transcriptome database. cDNA cloning primers (Table S3) were designed by the NCBI Primer-BLAST tool (https://www.ncbi.nlm.nih.gov/tools/primer-blast/index.cgi?LINK_LOC=BlastHome, accessed on 7 May 2023) according to the transcript sequence data of ‘Sorbonne’, and synthesized by Sangon Biotech (Sangon, Shanghai, China). The LohINT1 gene was amplified from the cDNA using PrimeSTAR^®^Max DNA polymerase (R045, TaKaRa, Dalian, China). Amplified PCR products were electrophoresed in 1% agarose gel and then purified with MiniBEST Agarose Gel DNA Extraction Kit Ver. 4.0 (9762, TaKaRa, Dalian, China). Sequencing of the PCR products was performed by Sangon Biotech (Sangon, Shanghai, China).

The transcript and the full-length CDS region of LohINT1 were amplified with primers LohINT1-GFP-F/R and LohINT1-YFP-F/R, respectively (Table S3). The amplified fragments were inserted into the Kpnl-linearized p221-GFP vacuole and EcoRI/Spel-linearized pUC-35S-YFP vacuole, respectively, by the seamless cloning method. Plasmids were transfected with maize yellowing seedling protoplasts prepared using the PEG-mediated method and observed with a confocal microscope (TCS SP8, Leica, Wetzlar, Germany) after overnight incubation. The cell membranes were labeled with an mCherry marker. Excitation/emission wavelengths for GFP, YFP, and mCherry were 488/(510–550) nm, 514/(525–575) nm, and 587/(607–650) nm, respectively.

### 4.7. Expression Analysis of Lily Sugar Transporter Gene

Expression in the transcriptome database was expressed as log^2^ transformed values of FPKM, and the expression value of each stage was the average of three biological replicates (Table S2). The data were normalized by Minitab version 20.3 and then the expression heat map was plotted using TBtools version 2.0 [78], which was used to identify the expression patterns of different sugar transporters during the initiation and development stage of bulblets.

### 4.8. Quantitative Real-Time PCR (qRT-PCR) Analysis

The total RNA of ‘Sorbonne’ and *Lilium* × *formolongi* were extracted from scale samples using an EASYspin Plus Complex RNA Kit (RN53 and RN40, Aidlab Bio, Beijing, China). RNA concentration was measured by NanoDrop2000 (Thermo Scientific, Waltham, MA, USA), and RNA quality was verified by 1% agarose gel electrophoresis using PowerPac^TM^ Basic (Bio-Rad, Hercules, CA, USA). Total RNA (1 μg and 1.6 μg) of each sample of ‘Sorbonne’ and *Lilium* × *formolongi* were reverse transcribed by the PrimeScript^TM^ RT reagent Kit with gDNA Eraser (RR047A, TaKaRa, Dalian, China) and PrimeScript^TM^ II 1st Strand cDNA Synthesis Kit with DNase I (6210A, TaKaRa, Dalian, China), respectively. Gene-specific primers were designed (Table S3) according to the previously described method (Section 4.6). Then, qRT-PCR was performed in a Bio-Rad ConnectTM optical module (Bio-Rad, Hercules, CA, USA) using the TB Green^TM^ Premix Ex Taq^TM^ kit (RR420A, TaKaRa, Dalian, China). All reactions were performed in three replicates in a 10 μL system at 95 °C for 2 min, followed by 40 cycles at 95 °C for 5 s and 60 °C for 30 s. The relative expression was calculated by the 2^−ΔΔCt^ method using *GAPDH* in the transcriptome of ‘Sorbonne’ and Unigene0053935_UBC22 in the transcriptome of *Lilium* × *formolongi* as the internal reference genes [79], and the correlation between transcriptome expression patterns and fluorescence quantitative results was compared separately.

### 4.9. Statistical Analysis

Values for three biological replicates were calculated as mean ± SEM. Differences between the groups were analyzed by one-way ANOVA with Duncan tests by SPSS Statistics version 17.0, and *p*-values less than 0.05 were considered statistically significant. Results were visualized by GraphPad Prism version 8.3.0.

## 5. Conclusions

In this study, we identified members of the sugar transporter family in the *Lilium* Oriental hybrid ‘Sorbonne’ and *Lilium* × *formolongi*. A total of 69 LohSTs and 41 LflSTs were found in the transcriptomes of ‘Sorbonne’ and *Lilium* × *formolongi*, respectively. Phylogenetic analyses showed that the MSTs could be categorized into seven subfamilies, SUTs into three subgroups, and SWEETs into four clades. According to the conserved motif analysis, different families of sugar transporters contain some essential or special conserved motifs, indicating that there are some functional differences among members of different families of sugar transporters. Conserved domain analysis showed that most SWEETs had two MtN3/saliva domains (also known as a PQ-loop repeat), which was significantly different from the single domain contained in MSTs and SUTs. Further expression analysis showed that 28 *LohSTs* were upregulated and expressed during the process of bulblet initiation, and 17 *LflSTs* were upregulated and expressed during the bulb swelling process that accompanies the transition of lilies from juvenile to adult. Finally, verified by qRT-PCR, we screened *LohSTP8*, *LohSTP12,* and *LflERD6.3* as key sugar transporter genes during lily bulb formation. Our study laid the foundation for elucidating the biological functions of sugar transporter genes in lily bulb formation.

## Figures and Tables

**Table 1:** Physicochemical properties and structural analysis of lily MSTs.

Name	Gene ID	Physicochemical Property							Second-Level Structure				Signal Peptide	TMD ^g^
		AA ^a^	MW ^b^	PI ^c^	II ^d^	AI ^e^	GRAVY ^f^	Subcellular Location	*α*-Helix	Extension Chain	*β*-Corner	Aperiodical Coil		
STP (sugar transport protein/hexose transporter)														
LohSTP1	Isoform_35954	508	56,512.57	9.75	39.44	108.62	0.380	Cell membrane	47.05	15.75	6.69	30.51	No	10
LohSTP2	Isoform_37643	518	57,031.77	9.13	34.51	108.05	0.498	Cell membrane	47.30	17.57	4.83	30.31	No	12
LohSTP3	Unigene0024941	507	55,105.69	9.00	35.58	107.32	0.585	Cell membrane	48.32	17.55	5.33	28.80	No	12
LohSTP4	Unigene0035034	520	56,842.79	8.98	39.92	107.62	0.559	Cell membrane	54.62	13.46	5.77	26.15	No	11
LohSTP5	Unigene0053898	524	57,736.85	9.19	40.79	106.77	0.463	Cell membrane	48.47	16.22	4.39	30.92	No	12
LohSTP6	Unigene0090674	511	56,022.08	8.80	39.65	111.82	0.537	Cell membrane	48.73	17.61	6.85	26.81	No	12
LohSTP7	Unigene011249	548	60,062.55	8.00	36.52	107.10	0.599	Cell membrane	50.73	15.33	5.29	28.65	No	13
LohSTP8	Unigene011258	523	57,544.02	9.31	32.02	105.72	0.610	Cell membrane	51.43	15.30	5.93	27.34	No	11
LohSTP9	Unigene011784	523	57,406.70	8.97	36.82	106.29	0.610	Cell membrane	51.24	16.83	4.78	27.15	No	12
LohSTP10	Unigene011878	494	53,558.23	8.92	38.19	117.59	0.717	Cell membrane	51.62	17.21	4.86	26.32	No	12
LohSTP11	Unigene012432	510	55,947.65	6.41	43.50	111.84	0.554	Cell membrane	50.98	15.69	5.69	27.65	No	10
LohSTP12	Unigene013245	503	55,493.61	9.09	34.43	109.68	0.624	Cell membrane	51.69	15.51	4.77	28.03	Yes	12
LohSTP13	Unigene015344	508	54,585.88	9.40	32.22	105.39	0.588	Cell membrane	48.62	16.93	4.72	29.72	No	11
LohSTP14	Unigene028908	266	30,490.10	8.70	36.42	103.98	0.507	Cell membrane	53.38	14.29	4.51	27.82	No	6
LohSTP15	Unigene25955 _L-Tis6-Transc	507	55,230.14	9.37	36.62	109.05	0.561	Cell membrane	47.93	16.57	5.33	30.18	No	12
LohSTP16	Unigene26933 _L-Tis6-Transc	313	35,346.22	9.51	50.90	111.73	0.592	Cell membrane	54.31	9.58	5.75	30.35	Yes	6
LflSTP1	Unigene0009642	524	56,408.33	8.82	32.24	99.90	0.462	Cell membrane	44.08	17.37	5.15	33.40	No	9
LflSTP2	Unigene0009644	366	39,004.88	10.19	38.82	112.98	0.620	Cell membrane	51.09	18.85	4.64	25.41	No	8
LflSTP3	Unigene0009691	506	55,597.37	8.33	43.20	110.81	0.536	Cell membrane	52.37	14.23	5.34	28.06	No	10
LflSTP4	Unigene0026021	493	53,656.28	9.45	36.54	113.94	0.655	Cell membrane	52.54	15.82	5.07	26.57	No	12
LflSTP5	Unigene0076409	500	54,447.33	9.50	36.74	110.00	0.596	Cell membrane	50.00	16.20	5.80	28.00	No	12
LflSTP6	Unigene0085933	522	57,288.83	9.53	34.37	103.12	0.594	Cell membrane	48.08	17.43	5.56	28.93	No	12
LflSTP7	Unigene0085934	536	58,873.19	7.61	38.36	108.40	0.601	Cell membrane	47.39	17.91	6.53	28.17	No	12
LflSTP8	Unigene0085935	326	36,501.98	8.16	51.56	97.45	0.225	Cell membrane	47.24	13.50	4.29	34.97	No	3
ERD6-like (early responsive to dehydration six-like)														
LohERD6.1	CL1228.Contig1 _L-Tis6-Transc	496	53,277.36	9.14	39.64	114.44	0.586	Cell membrane	53.23	53.23	7.46	21.98	No	12
LohERD6.2	Isoform_42958	471	50,332.01	8.35	34.14	109.30	0.624	Cell membrane	46.07	22.08	6.79	25.05	No	12
LohERD6.3	Unigene0027305	203	21,680.50	5.25	39.82	127.64	1.036	Cell membrane	52.71	22.17	5.42	19.70	No	6
LohERD6.4	Unigene0062718	486	51,640.58	8.41	41.32	111.60	0.651	Cell membrane	44.44	20.99	6.38	28.19	No	11
LohERD6.5	Unigene0085757	478	51,283.11	5.79	33.97	112.66	0.632	Cell membrane	48.54	19.87	5.65	25.94	No	12
LohERD6.6	Unigene009966	496	53,054.26	8.87	44.90	117.36	0.640	Cell membrane	49.60	19.56	7.26	23.59	No	12
LohERD6.7	Unigene0132508	490	52,929.09	7.46	28.93	114.16	0.652	Cell membrane	43.27	24.49	6.53	25.71	No	12
LohERD6.8	Unigene013892	481	51,582.69	8.32	31.62	111.50	0.680	Cell membrane	46.57	21.00	6.65	25.78	No	11
LohERD6.9	Unigene015457	470	51,168.34	6.36	35.34	108.23	0.631	Cell membrane	54.47	19.36	6.81	19.36	No	12
LflERD6.1	Unigene0030804	466	49,711.14	7.64	32.45	110.04	0.620	Cell membrane	47.64	21.67	6.44	24.25	No	11
LflERD6.2	Unigene0043193	502	54,520.94	8.94	42.79	97.11	0.295	Cell membrane	41.24	22.91	5.98	29.88	No	8
LflERD6.3	Unigene0080999	496	53,074.27	8.87	44.94	115.99	0.629	Cell membrane	49.60	19.56	7.26	23.59	No	12
LflERD6.4	Unigene0081000	400	42,864.09	9.17	41.62	114.57	0.534	Cell membrane	52.00	16.50	7.25	24.25	No	9
LflERD6.5	Unigene0084852	584	67,140.98	8.53	50.27	94.38	0.351	Cell membrane	22.09	40.41	8.73	28.77	No	8
INT (inositol transporter)														
LohINT1	Isoform_16749	285	30,965.09	5.47	45.11	107.82	0.400	Cell membrane	47.72	20.35	7.02	24.91	No	6
LohINT2	Isoform_25457	577	62,331.89	8.83	41.70	101.96	0.404	Cell membrane	43.67	18.37	4.68	33.28	No	12
LohINT3	Unigene006834	521	55,817.13	5.00	35.35	109.52	0.590	Cell membrane	49.90	18.62	5.37	26.10	No	12
LohINT4	Unigene008476	574	62,622.09	8.74	39.78	103.50	0.383	Cell membrane	43.73	18.64	4.53	33.10	No	12
LohINT5	Unigene010290	574	62,063.29	8.23	37.17	105.91	0.408	Cell membrane	45.30	18.12	5.57	31.01	No	12
LohINT6	Unigene0105364	404	43,968.26	7.40	47.06	96.04	0.259	Cell membrane	44.80	14.85	4.70	35.64	No	7
LohINT7	Unigene0130510	579	62,779.18	8.79	40.22	104.44	0.382	Cell membrane	42.66	18.48	5.18	33.68	No	12
LflINT1	Unigene0048321	569	61,606.12	8.92	41.85	102.04	0.417	Cell membrane	40.95	19.16	5.45	34.45	No	12
LflINT2	Unigene0060470	293	32,107.80	6.78	35.03	112.49	0.705	Cell membrane	55.29	14.68	4.78	25.26	No	7
LflINT3	Unigene0066618	574	62,073.28	8.23	36.20	105.73	0.403	Cell membrane	44.08	18.99	4.88	32.06	No	12
LflINT4	Unigene0066620	519	56,053.32	8.54	38.45	105.84	0.426	Cell membrane	42.00	18.69	5.39	33.91	No	10
pGlcT (plastidic glucose transporter)														
LohpGlcT1	Isoform_34139	483	51,883.10	8.74	32.69	113.02	0.652	Cell membrane	53.42	15.53	4.76	26.29	No	10
LohpGlcT2	Isoform_41236	300	32,479.90	8.55	40.63	105.33	0.496	Cell membrane	50.67	18.33	8.00	23.00	No	6
LohpGlcT3	Unigene007486	534	57,757.74	5.34	45.07	104.44	0.399	Cell membrane	48.13	14.79	5.24	31.84	No	9
LohpGlcT4	Unigene009251	534	56,342.16	9.46	35.05	112.32	0.586	Cell membrane	51.87	14.98	5.06	28.09	No	10
LohpGlcT5	Unigene0128566	265	28,883.13	8.73	39.97	112.23	0.618	Cell membrane	55.85	17.36	6.04	20.75	No	6
LohpGlcT6	Unigene26459 _L-Tis6-Transc	506	54,258.60	8.30	41.59	105.42	0.535	Cell membrane	53.75	13.44	5.73	27.08	No	10
LflpGlcT1	Unigene0042450	482	51,956.09	8.75	33.98	115.08	0.660	Cell membrane	56.43	13.90	4.98	24.69	No	10
LflpGlcT2	Unigene0067291	560	59,162.01	9.48	35.60	107.12	0.476	Cell membrane	51.07	15.71	6.79	26.43	No	10
LflpGlcT3	Unigene0074733	457	49,271.25	4.99	38.13	112.65	0.598	Cell membrane	56.02	14.44	5.25	24.29	No	10
PLT (polyol/monosaccharide transporter)														
LohPLT1	Isoform_38097	375	40,222.69	9.97	55.23	94.88	0.179	Cell membrane	37.60	13.07	6.67	42.67	No	5
LohPLT2	Unigene0039109	535	57,479.24	9.39	36.04	107.59	0.370	Cell membrane	49.72	15.14	5.23	29.91	No	10
LohPLT3	Unigene008609	499	53,758.00	7.65	40.55	111.78	0.575	Cell membrane	50.90	16.63	5.61	26.85	No	11
LohPLT4	Unigene012611	530	56,691.28	9.35	34.85	108.92	0.492	Cell membrane	50.00	14.91	5.09	30.00	No	11
LohPLT5	Unigene013635	512	54,854.36	9.75	35.74	109.61	0.510	Cell membrane	51.76	14.65	5.66	27.93	No	12
LohPLT6	Unigene0136537	510	54,679.07	5.77	46.96	117.75	0.603	Cell membrane	53.53	14.51	5.29	26.67	No	12
LflPLT1	Unigene0046661	525	56,159.61	9.27	35.02	108.84	0.506	Cell membrane	48.57	15.62	5.33	30.48	No	10
LflPLT2	Unigene0060312	521	56,049.54	8.70	40.88	110.42	0.551	Cell membrane	49.33	15.36	5.95	29.37	No	9
LflPLT3	Unigene0079147	506	54,175.45	5.60	48.60	118.87	0.626	Cell membrane	52.96	16.40	5.73	24.90	No	12
TMT (tonoplast sugar transporter)														
LohTMT1	Isoform_18721	386	42,178.39	4.80	59.26	73.50	−0.403	Cell membrane, Nucleus	25.65	14.51	2.59	57.25	No	1
LohTMT2	Unigene002282	749	80,237.27	5.20	43.78	104.22	0.363	Cell membrane	35.11	17.22	6.01	41.66	No	11
LohTMT3	Unigene003117	754	81,213.05	5.05	46.65	105.60	0.311	Cell membrane	35.68	16.45	5.70	42.18	No	11
LflTMT1	Unigene0030117	753	81,178.98	5.09	46.78	105.74	0.303	Cell membrane	36.12	17.00	5.98	40.90	No	10
LflTMT2	Unigene0030118	746	79,852.72	5.11	46.43	104.52	0.364	Cell membrane	34.85	17.02	5.90	42.23	No	10
VGT (vacuolar glucose transporter)														
LohVGT1	Unigene009635	551	58,545.62	9.14	41.93	119.55	0.579	Cell membrane	48.28	16.52	3.81	31.40	No	11
LohVGT2	Unigene26230 _L-Tis6-Transc	496	53,012.54	5.41	40.02	123.29	0.782	Cell membrane	52.42	17.34	5.04	25.20	No	12
LflVGT1	Unigene0055747	494	52,774.24	5.54	38.31	123.99	0.784	Cell membrane	47.98	19.43	6.07	26.52	No	12
LflVGT2	Unigene0057306	489	52,010.05	5.64	35.18	124.68	0.685	Cell membrane	49.28	19.02	5.11	26.58	No	11

^a^ Length of the amino acid sequence. ^b^ Molecular weight of the amino acid sequence, kDa is kilo Daltons. ^c^ Isoelectric point. ^d^ Instability index. ^e^ Aliphatic index. ^f^ Grand average of hydropathicity. ^g^ Number of transmembrane helices, as predicted by the TMHMM Server 2.0.

**Table 2:** Physicochemical properties and structural analysis of lily SUTs.

Name	Gene ID	Physicochemical Property							Second-Level Structure				Signal Peptide	TMD ^g^
		AA ^a^	MW ^b^	PI ^c^	II ^d^	AI ^e^	GRAVY ^f^	Subcellular Location	*α*-Helix	Extension Chain	*β*-Corner	Aperiodical Coil		
SUT2														
LohSUT3	Unigene006715	590	63,479.28	7.16	36.19	94.53	0.367	Cell membrane	35.59	16.27	4.75	43.39	No	11
LflSUT1	Unigene0027768	372	40,336.41	6.74	37.93	96.99	0.269	Cell membrane	35.48	15.05	3.76	45.70	No	5
SUT3														
LohSUT2	Unigene0016683	213	22,336.17	8.52	20.49	113.05	0.771	Cell membrane	43.19	20.19	7.51	29.11	No	5
LohSUT4	Unigene018685	270	29,486.60	9.17	28.87	102.89	0.440	Cell membrane	46.30	17.04	6.30	30.37	No	5
LohSUT5	Unigene021757	312	34,055.88	9.40	28.05	102.21	0.519	Cell membrane	55.77	16.03	5.77	22.44	No	6
SUT4														
LohSUT1	Isoform_39893	497	53,094.43	9.30	33.35	113.86	0.616	Cell membrane	44.47	16.50	4.02	35.01	No	12
LflSUT2	Unigene0057880	492	52,451.72	9.21	30.92	117.60	0.654	Cell membrane	46.14	14.43	3.66	35.77	No	12

^a^ Length of the amino acid sequence. ^b^ Molecular weight of the amino acid sequence, kDa is kilo Daltons. ^c^ Isoelectric point. ^d^ Instability index. ^e^ Aliphatic index. ^f^ Grand average of hydropathicity. ^g^ Number of transmembrane helices, as predicted by the TMHMM Server 2.0.

**Table 3:** Physicochemical properties and structural analysis of lily SWEETs.

Name	Gene ID	Physicochemical Property							Second-Level Structure				Signal Peptide	TMD ^g^
		AA ^a^	MW ^b^	PI ^c^	II ^d^	AI ^e^	GRAVY ^f^	Subcellular Location	*α*-Helix	Extension Chain	*β*-Corner	Aperiodical Coil		
Clade I														
LohSWEET2	CL5864.Contig1 _L-Tis6-Transc	251	27,820.21	9.66	31.32	111.04	0.639	Cell membrane	44.22	16.73	3.19	35.86	No	7
LohSWEET13	Unigene027020	230	25,686.81	9.15	42.13	119.48	0.887	Cell membrane	45.65	18.7	3.04	32.61	No	7
LflSWEET3	Unigene0035595	229	25,654.77	9.39	41.38	120	0.889	Cell membrane	45.85	19.21	3.93	31	No	7
LflSWEET8	Unigene0061686	151	16,952.45	10.01	35.97	118.68	0.874	Cell membrane	41.72	24.5	7.95	25.83	No	5
LflSWEET9	Unigene0061687	251	27,968.28	9.63	39.32	108.33	0.554	Cell membrane	45.42	20.32	3.98	30.28	No	7
Clade II														
LohSWEET9	Unigene019832	258	28,713.41	9.13	37.33	122.33	0.792	Cell membrane	37.6	22.87	3.88	35.66	No	7
LohSWEET11	Unigene024975	234	25,912.35	8.89	41.17	123.97	0.93	Cell membrane, Chloroplast	45.3	22.22	2.56	29.91	No	7
LohSWEET12	Unigene025150	257	28,632.48	9.26	35.63	129.26	0.808	Cell membrane	42.8	21.01	3.5	32.68	No	7
LflSWEET5	Unigene0056066	87	10,105.85	5.67	46.92	141.03	0.841	Cell membrane, Chloroplast	51.72	20.69	4.6	22.99	No	1
LflSWEET11	Unigene0081109	219	24,471.5	8.98	33.49	128.13	0.801	Cell membrane	33.33	21.92	2.74	42.01	No	6
LflSWEET12	Unigene0086230	215	24,143.01	9.27	33.61	125.02	0.838	Cell membrane	41.86	21.4	3.72	33.02	No	6
Clade III														
LohSWEET1	CL469.Contig2 _L-Tis6-Transc	272	30,486.59	8.98	42.67	125.04	0.891	Cell membrane	37.5	21.32	1.47	39.71	No	7
LohSWEET3	Unigene0026939	280	31,065.57	9.02	24.51	105.82	0.558	Cell membrane	39.64	16.07	2.86	41.43	No	7
LohSWEET4	Unigene0026940	254	28,783.35	7.59	28.72	113.9	0.65	Cell membrane	41.34	15.75	2.76	40.16	No	6
LohSWEET5	Unigene0026941	269	30,164.78	7.59	29	116.65	0.744	Cell membrane	43.87	18.22	2.97	34.94	No	7
LohSWEET6	Unigene0066766	331	36,516.11	5.29	40.76	114.53	0.58	Cell membrane	45.02	15.41	2.11	37.46	No	7
LohSWEET10	Unigene024768	277	31,108.8	8.63	28.83	110.87	0.631	Cell membrane	44.77	19.49	2.17	33.57	No	7
LohSWEET14	Unigene040101	194	21,758.8	8.61	42.85	120.52	0.436	Cell membrane	38.66	13.92	1.55	45.88	No	4
LohSWEET15	Unigene048317	123	14,174.51	6.56	49.08	89.43	−0.107	Chloroplast	30.08	14.63	1.63	53.66	No	2
LflSWEET1	Unigene0026980	256	28,865.72	9.1	44.97	123.71	0.905	Cell membrane	42.97	22.27	3.91	30.86	No	7
LflSWEET2	Unigene0026981	256	28,737.47	9.37	39.19	120.31	0.78	Cell membrane	41.8	22.66	3.12	32.42	No	7
LflSWEET6	Unigene0057475	263	29,746.2	8.34	33.34	112.28	0.644	Cell membrane	40.3	19.39	2.66	37.64	No	7
LflSWEET7	Unigene0059765	265	29,955.7	6.82	26.53	118.75	0.765	Cell membrane	36.6	20.38	3.4	39.62	No	7
LflSWEET10	Unigene0059765	77	8829.71	9.06	18.11	120.13	1.021	Chloroplast	36.36	36.36	2.6	24.68	No	2
Clade IV														
LohSWEET7	Unigene0122684	292	32,135.19	9.56	27.55	122.71	0.621	Cell membrane	46.58	15.07	5.14	33.22	No	7
LohSWEET8	Unigene0122684	243	27,016.7	8.45	31.94	107.45	0.514	Cell membrane	38.68	24.28	2.47	34.57	No	6
LflSWEET4	Unigene0046338	289	31,809.9	9.78	26.8	122.63	0.639	Cell membrane, Chloroplast, Peroxisome	42.91	15.92	3.81	37.37	No	7

^a^ Length of the amino acid sequence. ^b^ Molecular weight of the amino acid sequence; kDa is kilo Daltons. ^c^ Isoelectric point. ^d^ Instability index. ^e^ Aliphatic index. ^f^ Grand average of hydropathicity. ^g^ Number of transmembrane helices, as predicted by the TMHMM Server 2.0.

## Data Availability

Data is contained within the article and Supplementary Materials.

## Supplementary Materials

The following supporting information can be downloaded at https://www.mdpi.com/article/10.3390/ijms25063483/s1. Reference [80] is cited in the supplementary materials.
