# Supplementary material for: Identification and Expression Analysis of Putative Sugar Transporter Gene Family during Bulb Formation in Lilies
Source: Int J Mol Sci. 2024 Mar 20;25(6):3483. doi: 10.3390/ijms25063483 (PMC10970775; doi:10.3390/ijms25063483)
Supplement: Supplementary file 1 [file ijms-25-03483-s001.zip › Figure S1.pdf]

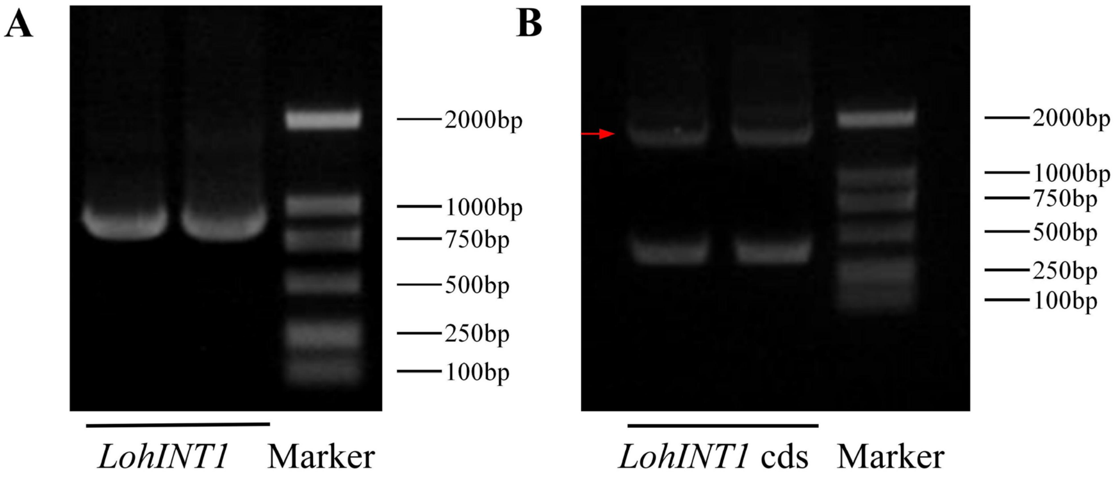

Figure S1: Results of 1% agarose gel electrophoresis analysis. (A) Agarose gel electrophoresis of *LohINT1* gene from transcript DNA. (B) Agarose gel electrophoresis of full-length *LohINT1* gene. The red arrow indicates the target band
